# The impact of vestibular schwannoma and its management on employment

**DOI:** 10.1007/s00405-021-06977-1

**Published:** 2021-07-04

**Authors:** O. M. Neve, J. C. Jansen, A. G. L. van der Mey, R. W. Koot, M. de Ridder, P. P. G. van Benthem, A. M. Stiggelbout, E. F. Hensen

**Affiliations:** 1 Department of Otorhinolaryngology and Head and Neck Surgery, Leiden University Medical Center, P.O. Box 9600, 2300 RC Leiden, The Netherlands; 2 Department of Neurosurgery, Leiden University Medical Center, Leiden, The Netherlands; 3 Department of Radiation Oncology, Leiden University Medical Center, Leiden, The Netherlands; 4 Department of Biomedical Data Sciences, Leiden University Medical Center, Leiden, The Netherlands

**Keywords:** Vestibular schwannoma, Employment, QoL, Surgery, Radiotherapy, Surveillance

## Abstract

**Background:**

Employment is an important factor in quality of life. For vestibular schwannoma (VS) patients, employment is not self-evident, because of the sequelae of the disease or its treatment and their effects on daily life.

**Objectives:**

This study assessed employment status, sick leave (absenteeism) and being less productive at work (presenteeism) in the long-term follow-up of VS patients, and evaluated the impact of treatment strategy (active surveillance, surgery or radiotherapy).

**Methods:**

A cross-sectional survey study was performed in a tertiary university hospital in the Netherlands. Patients completed the iMTA-post productivity questionnaire (iPCQ). Employment status was compared to that of the general Dutch population. Employment, absenteeism and presenteeism were compared between patients under active surveillance, patients after radiotherapy and post-surgical patients.

**Result:**

In total 239 patients participated, of which 67% were employed at the time of the study. Only 14% had a disability pension, which was comparable to the age-matched general Dutch population. The proportion of patients with absenteeism was 8%, resulting in a 4% reduction of working hours. Presenteeism was reported by 14% of patients, resulting in a 2% reduction of working hours. The median number of working hours per week was 36, and since the diagnosis, these hours had been reduced by 6%. There were no significant differences between treatment modalities.

**Conclusion:**

On average, long-term employment status and working hours of VS patients are comparable to the age-matched general population. Treatment strategies do not seem to differentially impact on long-term employment of VS patients.

## Introduction

Despite the benign character of vestibular schwannomas (VS), the tumor can have a substantial impact on patients’ lives. Typically, the tumor causes hearing loss, tinnitus and balance disorders. However, headache, facial numbness, facial pain or facial paresis may also occur [1]. These symptoms can reduce health-related quality of life (HRQL), and VS patients have reported even worse HRQL than patients with chronic diseases or head and neck cancer [2].

Multiple treatment options exist for sporadic VS, which can be subdivided in three broad categories: active surveillance, radiotherapy and surgery. The treatment of choice depends on tumor characteristics (i.e., tumor size, progression), patient characteristics (i.e., age, symptoms, patient preference) and probably also on other factors such as availability in the particular hospital. The aim of all three strategies is tumor control, but the way this is achieved differs: with surgery the tumor mass is removed, either totally, near totally or subtotally [3]. Radiotherapy is aimed at arrest of tumor progression, while the tumor remains in situ. Active surveillance does not intervene with the tumor, but relies on the observation that a small majority of VSs does not show progression once detected [3]. In this strategy, active therapy is reserved for progressive disease. Importantly, none of these treatment options is well suited for curing hearing or vestibular function loss, and all confer a risk to hearing, balance, trigeminal or facial nerve function. As VS in general is not a life-threatening disease when adequately managed, most patients will live with their tumor, the associated symptoms and/or the sequelae of therapy for prolonged periods of time. As such, a VS can be viewed as a chronic disease and the impact on HRQL may, thus, be lifelong. Because of this, HRQL is one of the guiding principles in VS management.

An important aspect of HRQL that is often overlooked is a patient’s ability to participate in professional life [4]. In VS patients, professional performance is not self-evident and may be impacted by associated symptoms and the reported social restrictions that patients experience because of them [5, 6]. A patient’s ability to acquire or maintain a job is critical from both a societal and personal perspective. Being employed is not only of paramount importance from an economic perspective, but also associated with increased self-esteem and self-worth [7]. Furthermore, work provides relationships, social connections and a higher level of social status, and having secure employment helps people to prevent developing illness [8–10]. Conversely, being unemployed is associated with a poorer physical and mental health [9].

In previous studies, VS patients’ employment rates varied between 45 and 80% [11–14]. Most studies have assessed the employment rate pre- and post-surgery or radiotherapy, and only one has assessed employment rates in VS patients under active surveillance. In addition to employment rates, the reduced productivity while having a job (presenteeism) and sick leave from a job (absenteeism) are important additional aspects of professional participation that have not yet been investigated in VS.

This study assesses the long-term effects of VS on employment and productivity and analyzes the long-term impact on employment of surgery, radiotherapy and active surveillance. Furthermore, determinants of unemployment and reduced productivity are evaluated.

## Methods

This cross-sectional study was part of a more extensive study on long-term VS outcomes in the Netherlands. Participating patients from a questionnaire study in 2014 were reapproached for participation [15]. All patients were diagnosed with unilateral VS between 2003 and 2014. Patients were diagnosed and/or treated at Leiden University Medical Center, an expert center for VS offering different management options, including active surveillance, surgery (mostly through translabyrinthine or retrosigmoidal approach) and fractionated stereotactic radiotherapy. Patients who prefer radiosurgery were referred to another clinic.

For this study, all patients between 18 and 67 years were included, since the retirement age is currently elevated stepwise from 65 to 67 years between 2013 and 2024 in the Netherlands. Exclusion criteria were other skull base pathologies and insufficient proficiency in the Dutch language to complete the questionnaires. After providing informed consent, patients could complete questionnaires electronically or on paper between April and September 2020.

### Questionnaires

Employment status was assessed using the Productivity Costs Questionnaire (iPCQ). The iPCQ measures productivity loss from the societal perspective and contains three different modules: absenteeism, presenteeism, and productivity loss in unpaid/volunteer work. The first two modules are validated and the validation of the productivity loss for unpaid work is still in progress [16]. Productivity losses were calculated in hours following the manual [16]. The recall period used in the questionnaire is 4 weeks [16]**.** In addition, questions about productivity before diagnosis were asked, while recognizing the reduced reliability due to the prolonged recall period.

Sex, age, time since the treatment and tumor size at diagnosis were acquired from the electronic patient records. Tumor size was scored at diagnosis using the reporting system proposed by Kanzaki et al. [17]. The definition of Statistics Netherlands (CBS) for low, middle and high education level was used, which follows the international standard classification of education [18]. Frequencies were calculated for categorical variables and means and standard deviation (sd) for normally distributed numerical variables and medians and interquartile ranges (IRQ) for not-normally distributed numerical variables. Baseline characteristics of responders and non-responders were checked in a non-responder analysis.

Employment status of patients aged 45–65 years was compared to the general Dutch population aged between 45 and 65 years using a chi-squared test. This age category was the best matched age group to the study population available in the public data of Statistics Netherlands [19]. Employment status per treatment modality was compared using logistic regression. Sex, age, and educational level were included in the regression to correct for potential confounding. The goodness of fit was checked with a model Chi-squared test.

Absenteeism and productivity in hours were compared to the general Dutch population in the third quarter of 2020. The effect of treatment modalities on productivity in hours per week was assessed in a linear regression. Sex, age and educational level were included to correct for potential confounding. Model assumptions were visually checked. The differences pre- and post-diagnosis of productivity in hours per week were analyzed per treatment modality.

All analyses were performed using SPSS version 26 (IBM SPSS Inc., Armonk, USA). A *p* value < 0.05 was considered statistically significant. Incomplete questionnaire modules (absenteeism, presenteeism, unpaid work) were excluded from the analysis. According to our power calculation, a minimum number of 37 participants per group were needed to detect a difference in the productivity of 8 h in 4 weeks (*α* = 0.05, 1 − *β* = 0.8, assuming *σ* = 6).

## Results

**Fig. 1:**
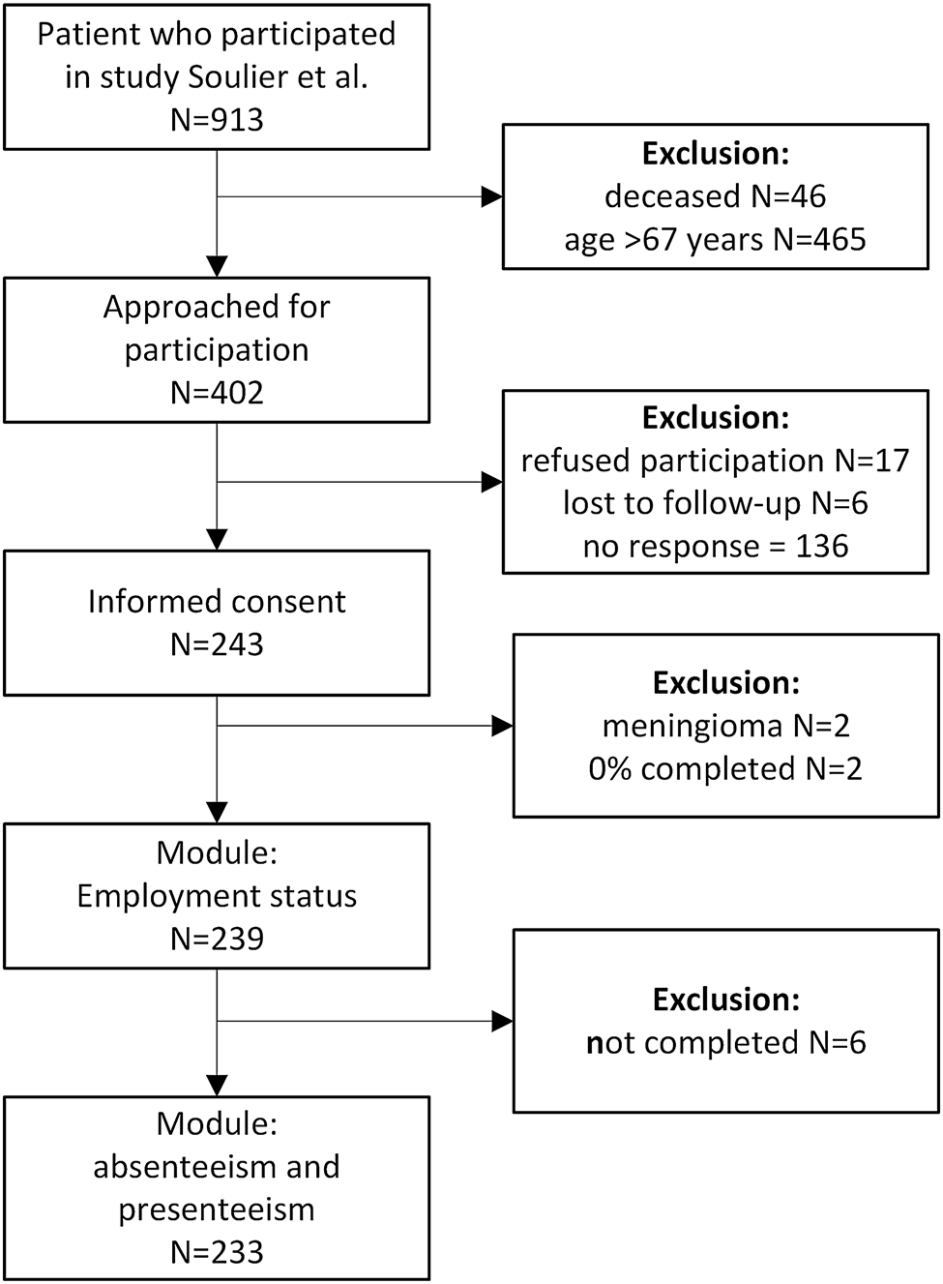
Flowchart. Patients who participated in a survey study in 2014 were reproached for participation in this study. Patients aged > 67 years (retirement age in the Netherlands) were excluded. Two patients had a different diagnosis after pathology and were excluded

In total, 402 patients were approached for participation, of whom 243 (60%) provided informed consent. There were no significant differences between responders and non-responders regarding age, sex and educational level. In the responder group, two patients were excluded because histology showed meningioma rather than schwannoma and two patients did not complete any questions after providing informed consent (Fig. 1).

As shown in Table 1, the study population consisted of 95 patients under active surveillance, 113 post-surgery patients, 24 post-radiotherapy and seven patients who underwent both surgery and radiotherapy over the years. Post-surgical patients were younger and had a larger tumor size at diagnosis compared to patients who underwent radiotherapy or surveillance, most likely a reflection of the indications for surgery in The Netherlands.

**Table 1:** Baseline characteristics

	Total		Active surveillance		Surgery		Radiotherapy		Surgery and radiotherapy	
	*N* = 239		*N* = 95		*N* = 113		*N* = 24		*N* = 7	
	*N*	%	*N*	%	*N*	%	*N*	%	N	%
Age										
< 45 yrs	14	6	3	3	9	8	1	4	1	14
45–54 yrs	49	21	18	19	24	21	4	17	3	43
55–64 yrs	135	57	61	64	58	51	15	63	1	14
65–68 yrs	41	17	13	14	22	20	4	17	2	29
Sex (male)	125	52	55	58	54	48	12	50	4	57
Educational level										
Low	60	25	21	22	30	27	6	25	3	43
Middle	82	34	36	38	36	32	7	29	3	43
High	97	41	38	40	47	42	11	46	1	14
Kanzaki at diagnosis										
Intrameatal	73	31	48	51	17	15	7	29	1	14
Small (0–10 mm)	55	23	22	23	26	23	6	25	1	14
Medium (11–20 mm)	59	25	25	27	25	22	9	38		
Moderately large(21–30)	32	13			28	25	2	8	2	29
Large (31–40 mm)	13	5			11	10			2	29
Giant (> 40 mm)	7	3			6	5			1	14
Time in years (median)										
Since treatment (IQR)	10	(8–12)	9	(8–11)	11	(9–14)	8	(6–9)	9	(7–13)

The baseline characteristics of all participants are shown. The right column shows the patient who underwent both surgery and radiotherapy since diagnosis. Kanzaki represents the classification of Kanzaki et al. of the tumor size at diagnosis

*Yrs *years, *IQR* interquartile range

### Employment status

**Fig. 2:**
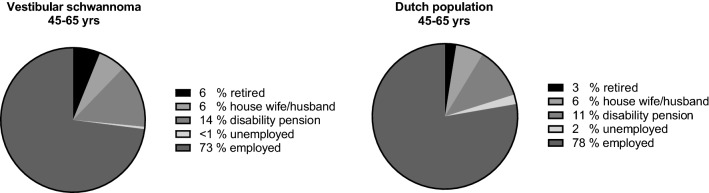
Employment status. The employment status of vestibular schwannoma patients aged 45–65 years (left) is compared to the reference population in the Netherlands (right). Patients who voluntarily do not have paid employment are labeled as ‘house wife/husband’

Figure 2 shows the employment status of the study population aged between 45 and 65 years (*N* = 196) and the Dutch population aged 45–65 years. Patients < 45 years (*N* = 14) and 66–67 (*N* = 28) were excluded from this comparison. Unemployment and voluntary unemployment (e.g., housewife or husband) were comparable in both groups. VS patients were more often retired compared to the general Dutch population (*χ*^2^, *p* value 0.006), although the size of the differences is rather small: retirement was found in 6% vs. 3%, respectively, and disability pensions in 14% vs. 11%, respectively, resulting a slightly lower proportion of employment for VS patients (72% vs. 78%, respectively).

Unemployed VS patients were on average 3 years older, more often female (64% vs. 41%), and more likely to have a low level of education (37% vs. 19%) than employed patients. There were no differences between employed and unemployed patients for treatment modality or time since treatment.

The probability of being employed was assessed per treatment modality using logistic regression, as shown in Table 2. Model assumptions were met, although the explained variance of both models was relatively low. There were no differences between patients under active surveillance, after radiotherapy or after surgery. We found a tendency for a higher risk of unemployment in patients who underwent both surgery and radiotherapy. However, due to the small number of patients in this group, this was not statistically significant.

**Table 2:** Logistic regression assessing the effect of treatment modality on employment status (yes/no)

	Model 1^a^		Model 2^b^	
	OR	CI 95%	OR	CI 95%
Active surveillance (reference)	–		–	
Surgery	0.97	0.53;1.78	1.04	0.54;1.98
Radiotherapy	1.01	0.38;2.71	1.06	0.38;3.01
Surgery and radiotherapy	0.55	0.16;2.64	0.44	0.08;2.57
Sex (female)			0.37	0.23;0.62
Age			0.83	0.81;0.86
Educational level				
Low			0.66	0.36;1.21
Middle			1.02	0.58;1.79
High (reference)			–	
*R*^2^	0.002		0.11	
*χ*^2^	0.003		< 0.001	

*OR* odds ratio, *CI* confidence interval

^a^Treatment included

^b^Treatment, sex, age and educational level included.

### Absenteeism and presenteeism

The number of working hours per week and the productivity losses due to absenteeism and presenteeism for employed VS patients (*N* = 160) are shown in Table 3. Overall, the weekly working hours of VS patients did not differ significantly from the general Dutch employed population. In the active surveillance group, the working hours per week seemed slightly higher, but this difference disappeared after correction for confounding factors such as age, educational level and sex, as shown in the linear regression shown in Table 4.

**Table 3:** Absenteeism and presenteeism

	Total	Active surveillance	Surgery	Radiotherapy	Dutch population^a^
	*N* = 160	*N* = 65	*N* = 74	*N* = 17	
Hours/week					
Median (IQR)	36.0 (24–40)	36.0 (28–40)	32.0 (24–40)	36.0 (22–40)	
Mean (sd)	32.6 (12.1)	34.7 (10.4)	30.4 (10.5)	32.4 (14.9)	31
Difference pre diagnosis	− 6.2%	− 4.8%	− 8.0%	− 5.6%	
% Presenteeism	1.8%	1.1%	2.6%	1.3%	
% Absenteeism	4.2%	4.3%	4.3%	0%	4.4%

All patients we were employed are included in this table. Per treatment modality the working hours and productivity loss due to presenteeism and absenteeism are shown. There were four employed patients who underwent both surgery and radiotherapy, because of this small sample size they are not included as separate group in this table

^a^Dutch population statistics from 3rd quarter of 2020 obtained from Statistics Netherlands

**Table 4:** Linear regression assessing the effect of treatment modality on working hours per week

	Model 1^a^		Model 2^b^	
	Coefficient	95% CI	Coefficient	95% CI
Active surveillance (reference)	–		–	
Surgery	− 4.0	− 7.73;− 0.35	− 2.9	− 6.17;0.29
Radiotherapy	− 2.2	− 8.13;3.37	− 1.5	− 6.68;3.70
Surgery and radiotherapy	4.1	− 7.07;15.35	2.4	− 7.64;12.47
Sex (female)			− 11.33	− 14.82;− 7.85
Age			− 0.21	− 0.41;0.00
Educational level			0.22	− 1.98;2.42
R2	0.037		0.29	
Model ANOVA	0.12		< 0.001	

Educational level was categorized as 1,2,3, for low, middle and high level, respectively

^a^Treatment included

^b^Treatment, sex, age and educational level included

In addition to the working hours, the proportion of absence due to sick leave (absenteeism) was comparable to the sick leave rates in the general Dutch population, just over 4% of the total worked hours. Thirteen patients (8%) mentioned absenteeism in the last 4 weeks, causing an average of 17 h of productivity loss in 4 weeks. Of these 13 patients, seven had been absent for the entire period of 4 weeks.

Furthermore, 23 patients (14%) mentioned that they were less productive while being at work. The extent of this presenteeism was low across all treatment modality groups, with a percentage of just under 2% of all worked hours. The productivity loss of these 23 patients were on average 3.2 h per 4 weeks.

One out of six patients reported that their working hours have changed since the diagnosis. Of these 40 patients, 34 patients worked fewer hours and six worked more hours per week. Overall, the working hours decreased with 6%. Differences between treatment modality groups were statistically significant but small, with a decrease in working hours of 8%, 6% and 5% for surgery, radiotherapy and active surveillance, respectively (*χ*^2^, *p* < 0.001).

## Discussion

This study shows that employment status of patients with VS on average is quite comparable to a reference group of the general Dutch population. In addition, differences in sick leave rates (absenteeism) also were not statistically significant. Some patients however did report productivity losses while being at work (presenteeism). Employment rate, sick leave rate and productivity loss did not differ between the treatment modalities (surgery, radiotherapy and active surveillance), with the possible exception of patients receiving both radiotherapy and surgery.

Previous research on employment in VS patients described the differences pre- and post-treatment. Post-surgery, the percentage that could maintain their employment varied from 69 to 80% [13, 14, 20]. One of these studies reported that 79% of VS patients under active surveillance maintained their paid job [20]. Another small study on radiotherapy in young (< 40yrs) VS patients reported that all patients maintained their employment [12]. In the current study, 15% of the patients reported a reduction in working hours after the diagnosis. The decrease in working hours (− 8%) was the largest in the surgery group, followed by radiotherapy (− 6%) and active surveillance (− 5%). These differences are small and as the reduction was not necessarily due to illness or therapy related factors, causality is unclear. Even so, active treatment most likely will impact on working hours in the recovery phase directly after treatment. For example, patients who undergo surgery are expected to have higher absenteeism rates in the direct postoperative phase. However, the results of this study indicate that this effect is temporary and that treatment modality does not differentially impact on long-term employment rates of VS patients, in contrast to age, sex and education level.

Employment status was compared with the Dutch population aged 45–65 years. This comparison showed that the proportion of disability pensions was almost similar. This finding contrasts with a Norwegian study, in which a threefold higher proportion of disability pensions (22%) was reported compared to the general population (6%) [11]. This difference may be explained by the choice of reference population in the Norwegian study, since their reference group seemed to be the total Norwegian working-age population (i.e., all age groups). In our study, we opted for using the Dutch general population between 45 and 65 years as reference group because this matched the age distribution of the VS patients in this study most closely. As older people are more likely to have disability pensions, comparing with an age-matched population seems reasonable.

We found that the proportion of retired persons was mildly larger in the VS patient group than in the reference group. This difference is probably due to a slightly different age distribution in the reference and study populations, as patients aged 65 are relatively overrepresented in the latter. However, it is also conceivable that patients opt for early retirement due to sequelae of VS or its treatment.

Absenteeism (i.e., the number of working hours lost due to illness) in VS patients was similar to the entire Dutch working-age population in the third quarter of 2020, when the questionnaires were completed. Absenteeism was less prominent in the VS patients than in pituitary tumor or irritable bowel syndrome patients (8% vs. 40% vs. 34%) [21, 22]. For presenteeism, no reference from the general population was available. However, the presenteeism incidence was lower than in patients with pituitary tumors (14% vs. 39%) and irritable bowel syndrome (14% vs. 61%).

This study has some inherent limitations. The cross-sectional design of the study precludes causal inferences. In addition, radiotherapy was underrepresented in the study and below the required number needed for a power of 80%. This could lead to type II errors in which real differences in employment after radiotherapy were not identified. Furthermore, patients were asked about their employment situation before the diagnosis. As a consequence of the extended follow-up, the time of diagnosis exceeded the recommended recall period of 4 weeks ago. This prolonged period yields a risk of recall bias and the results of employment pre- and post-diagnosis should be interpreted with care. Last, the study was performed in the Netherlands, a country with an extensive social security system. It might be possible that employment rates are lower than in countries with a more limited social security system. The setting of this study should be considered when translating the results to other countries.

Strengths of the study include the relatively large cohort of VS patients and their long-term follow-up (median 10 years). In addition, all three treatment modalities were included allowing analysis of their differential effect on both employment status and productivity. Furthermore, this is the first study that assessed absenteeism and presenteeism in VS patients.

## Conclusion

This study suggests that long-term employment in VS patients on average is comparable to the employment in the general population, regardless of the treatment modality. There were no differences between sick leave and disability rates of VS patients and the age-matched general population. Although absenteeism is variable in VS patients and increased absenteeism may be expected shortly after active VS treatment, the results of this study indicate that the long-term prospects for the employment of VS patients in general are encouraging, irrespective of the treatment strategy. This information is valuable in counseling and medical decision making.

## Data Availability

The dataset analyzed during the current study is not publicly available but is available from the corresponding author on reasonable request.
